# Pennsylvania Hospital

**Published:** 1838-01-03

**Authors:** 


### CLINICAL REPORTS.

### PENNSYLVANIA HOSPITAL.

*Case of double hare-lip in an adult; operation on both sides at the same time, by Dr. Randolph; cure in three weeks.*

A farmer, residing in Virginia, aged 25 years, was admitted into the Hospital, with this congenital malformation, on the 22d of November, 1837. In addition to the double fissure of the lip, there was a division of the bony palate throughout its whole extent, the bones being separated three lines from each other, and the two parts of the uvula drawn six lines asunder. The isolated portion of the lip, together with the two central incisors, and the portion of the alveolar processes joined to them, was attached to the cartilaginous prolongation of the vomer, so that the point of the nose was drawn closely towards these teeth, which were rendered useless from an unnatural projection. He also laboured under very indistinct articulation. Owing to prejudices on the part of his parents, no operation had been permitted during childhood.

On the 29th of November, he was introduced into the amphitheatre of the Hospital. Dr. Randolph, in noticing the case, remarked that the operation for hare-lip was occasionally attended with danger, and instanced some cases reported by Sir Astley Cooper, in which the operation on infants had terminated fatally. It is therefore now rarely performed under the age of eighteen months or two years. In cases of double hare-lip most surgeons recommend that the operation be practised on one side at a time. The present subject, however, said Dr. Randolph, from his age, good health, and the large size of the central portion, is a fit case for a simultaneous operation on both sides. Besides the removal of a disgusting deformity, the articulation of the patient will be much improved, as well as his capability of taking liquids into the mouth. Bony union is not to be expected; though, when the operation is performed on children, that result not unfrequently takes place. From the rigidity of the soft palate, in this case, no benefit will be likely to arise from a future performance of staphyloraphy.

In this operation some surgeons use the knife. Dr. R. prefers a pair of scissors. Having dissected the edges of the lip from its attachment to the gums, for a short distance on each side, he cut away the external edges of the fissures, and with a small scalpel pared off each side of the central portion. Two long silver needles, armed with steel points, were then passed through the three parts; the usual figure of eight ligature was applied, and the cut surfaces drawn into contact. The patient was directed not to speak, and liquids ordered for diet.

December 3d. The upper needle was removed, leaving the ligature undisplaced; on the 4th, the second needle was taken away, the ligature still adhering, by means of the coagulated blood. On the 8th, these were taken away; when the edges of the lip were seen to have united completely, by the first intention: the projection of the teeth producing some pressure on the cicatrix, a piece of Mahy's plaster was interposed between them and the lip. The patient was allowed the usual diet of the house.

Dec. 13. Being anxious to have an artificial palate, he was seen to-day by an eminent dentist of the city, who dissuaded him from it, as his articulation is now sufficiently distinct, and his power of swallowing very much improved; he was of opinion that if a plate were applied to the roof of the mouth, it might separate the bones from each other, so as in time to prove a serious inconvenience. In the evening the lip was attacked with erysipelas, which was prevailing in the house to a great extent. He was ordered a dose of salts and magnesia, to keep the part constantly wet with cold leadwater, and his diet to be reduced to gruel.

Dec. 15. The erysipelas having disappeared, and the pressure of the teeth having produced some slight ulceration, they were cut off with a pair of strong bone-nippers, just above the gums.

The man was discharged on the 20th, perfectly cured.

---

*List of accidents admitted into the Pennsylvania Hospital, from Dec.* 13*th to Dec.* 27*th*, 1837.

One fracture of the forearm; one fracture of the clavicle; two fractures of the tibia and fibula; one fracture of the fibula, an inch and a half above the ankle joint, with very little displacement; one case of fracture of the metatarsal bone, accompanied with great contusion, with a lacerated wound in the sole of the foot, caused by a locomotive; one case of concussion of the spine; an incised wound of nose and lip; a case of frosted finger in a black sailor.

A severe compound comminuted fracture of the skull, caused by the bursting of a gun, was admitted on the afternoon of Christmas. The dura mater was laid bare in a line of three inches, and for the width of half an inch. Three large spiculæ of bone were removed, and the edges of the wound slightly drawn together, leaving a free vent for the discharge, and a cold poultice applied. V. S. ad. f℥viii. Barley Water. Died on the morning of the 27th.

